# Sometimes when you hear hoof beats, it could be a zebra: consider the diagnosis of Fabry disease

**DOI:** 10.1186/1471-2369-13-73

**Published:** 2012-07-31

**Authors:** James O Burton, John P Dormer, Helen E Binns, Warren P Pickering

**Affiliations:** 1 Department of Infection, Immunity & Inflammation, School of Medicine and Biological Sciences, University of Leicester, Leicester, LE1 9HN, UK; 2 Department of Histopathology, University Hospitals of Leicester NHS Trust, Leicester, UK; 3 Department of Cardiology, Northampton General Hospital, Northampton, UK; 4 Department of Nephrology, Northampton General Hospital, Northampton, UK

**Keywords:** Anderson-Fabry disease, Renal biopsy, Zebra bodies, Multi-system disease

## Abstract

**Background:**

Fabry disease is an X-linked lysosomal storage disorder that results from a deficiency of the enzyme α-galactosidase A. Fabry disease is present in 4–5% of men with unexplained left ventricular hypertrophy or cryptogenic stroke. As enzyme replacement therapy is now more widely available, it is important to recognise the signs and symptoms of the disease and establish the diagnosis so that early treatment can be started before irreversible organ damage occurs.

**Case Presentation:**

A previously fit and well 32-year-old Caucasian male presented with multisystem dysfunction including renal impairment. Although he had no suggestive symptoms, a diagnosis of Fabry disease was first established on a native renal biopsy. This was confirmed by enzymatic testing and subsequent genetic analysis that revealed a potentially new pathogenic variant.

**Conclusions:**

This case highlights the importance both of Fabry disease as a differential diagnosis in patients with renal impairment in the context of multi-system disease and also of adequate tissue sampling for electron microscopy when performing native renal biopsies.

## Background

Fabry disease is a rare x-linked lysosomal storage disorder. However, with new diagnostic techniques it is being increasingly recognised as a cause of end-stage renal disease. With the emergence of enzyme replacement therapies, early diagnosis of this multisystem disease is crucial. We describe a patient who presented with non-specific symptoms whose diagnosis of Fabry disease was established on a native renal biopsy.

## Case Presentation

**Figure 1:**
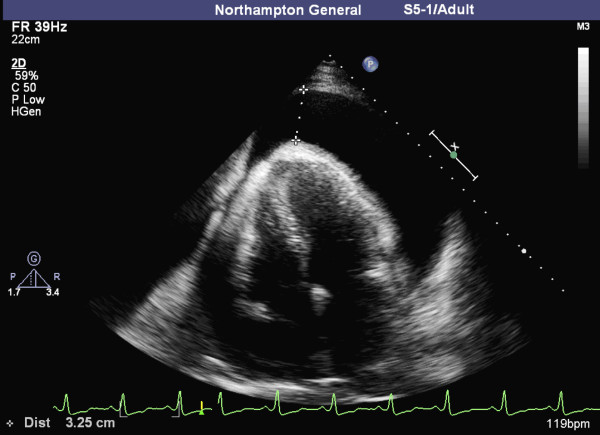
Apical four-chamber ECHO demonstrating: a 3.5 cm pericardial effusion apically (at the cursor) and more than 4 cm around the anterior wall; right atrial collapse from resulting tamponade and; although there was no evidence of left ventricular hypertrophy, the highly echogenic myocardium is also typical of Fabry's.

A 32-year-old Caucasian male presented with a three-week history of fever, abdominal pain, cough and exertional breathlessness after a recent trip to Turkey. He had been previously entirely healthy with no significant family history of note and was taking neither regular nor recently prescribed medications. He had no history of hypertension and his haemodynamic parameters remained normal throughout his admission. Investigations revealed: a white cell count of 13.8 x10^9^/L; C-reactive protein of 278 mg/L; alanine transaminase of 468 units/L; serum albumin of 30 g/L and a serum creatinine of 535 μmol/L. There was evidence of haematoproteinura on dipstick urinalysis with a protein to creatinine ratio of 285 (normal range <30). A chest x-ray showed no evidence of consolidation, but revealed significant cardiomegaly. A subsequent echocardiogram revealed a bright echotexture to the myocardium and confirmed the presence of a 4 cm global pericardial effusion causing right atrial collapse, the cardinal sign of cardiac tamponade (Figure 1). An ultrasound scan of his abdomen demonstrated splenomegaly without hepatomegaly, and kidneys of normal echotexture, the right measuring 9 cm and the left 10.3 cm. A nephritis screen including serum ANA, ANCA, complement levels and immunoglobulins was unremarkable.

He was treated initially for an atypical infection with appropriate broad-spectrum antibiotic cover with a corresponding improvement in both white cell count and CRP levels. The day after admission he underwent a pericardiocentesis because of the risk of tamponade. Subsequent histological examination of the pericardial fluid showed evidence of acute on chronic pericarditis secondary to uraemia. All cultures of blood, urine and pericardial fluid (including mycobacterium, leptospirosis and mycoplasma) were negative. Despite these measures, his creatinine continued to rise and a renal biopsy was performed.

**Figure 2:**
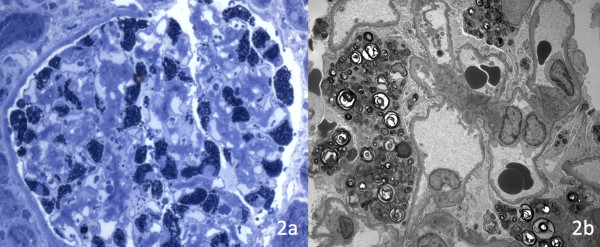
Histological sections showing: a) toluidine blue staining demonstrating abundant deposition of glycolipid inclusions within the podocytes and; b) electron microscopy with ‘zebra’ bodies - enlarged secondary lysosomes packed with lamellated membrane structures.

Light microscopy showed: very active tubulointerstitial inflammation with lymphocytic and plasma cell infiltrates as well as interstitial oedema related to his concurrent antibiotic treatment with intravenous co-amoxiclav nd; global sclerosis of all 22 sampled glomeruli. Toluidine blue staining revealed abundant deposition of glycolipid inclusions within the podocyte (Figure 2a). Immunofluorescence was negative. Electron microscopy demonstrated the characteristic enlarged secondary lysosomes (myeloid or ‘zebra’ bodies) packed with lamellated membrane structures that are associated with Fabry disease (Figure 2b). The diagnosis was later confirmed, initially by a blood spot α-galactosidase activity of 3.61 pmol/spot/h (5.51-58.1) and subsequently by a reduced plasma α-galactosidase A activity of 1.88 μmol/L/h (3-20) as well as elevated levels of globotriaosylceramide (Gb3) in the urine at 0.5 mg/mmol creatinine (0-0.03). Genetic sequence analysis revealed a missense point mutation causing the substitution of arginine with proline at amino acid residue 356, consistent with a diagnosis of Fabry disease.

On resolution of his sepsis, his C-reactive protein and alanine transaminase levels returned to normal and a repeat abdominal ultrasound confirmed that his splenomegaly had also resolved. Despite an initial improvement in serum creatinine to 250 μmol/L after commencement of steroid therapy for the underlying antibiotic-induced nephritis, he became dialysis dependent after six months, although subsequently received a living, non-related renal transplant from his wife. He is now receiving enzyme replacement therapy.

Interestingly, he had no symptoms suggestive of Fabry disease. In particular there were no acroparathesiae, gastrointestinal symptoms and no history of heat intolerance; he described sweating normally on exertion. He also denied any problems with his skin.

## Discussion

Fabry disease is caused by mutations in the gene encoding the lysosomal enzyme α-galactosidase A [1]. This results in reduced or absent α-galactosidase A activity and intra-lysosomal accumulation of neutral glycosphingolipids, mainly Gb3 (a substrate of α-galactosidase A), in many cells including renal epithelial cells, endothelial cells, vascular smooth muscle cells, cardiac myocytes and neurons of the autonomic nervous system. As Gb3 is easily accessible in both plasma and urine and seems to be directly involved in the renal pathology of Fabry disease, it may be a potential diagnostic assay for patients presenting with the classical disease phenotype [2]. However, its exact utility in the diagnosis and prognosis of Fabry remains contentious [3].

Interestingly, the genetic sequence analysis in this case revealed a hemizygous mutation for a G to C transversion at nucleotide 1067 (c.1067 G > C) in exon 7 of the α-galactosidase A (GLA) gene. This variant is predicted to result in the substitution of arginine with proline at amino acid residue 356 (p. Arg356Pro). Although not previously described in the literature, two mutations have been reported in Fabry patients that cause substitution of the same Arg356 residue (p. Arg356Trp and p. Arg356Gln) [4,5]. Both mutations produce small amounts of residual enzyme (as in this case), but still at levels that would cause disease. Therefore, in the context of all the other clinical findings, this variant is considered to be pathogenic.

Estimates of the incidence of Fabry disease vary markedly and although rare, it is likely to be more common than originally thought. A newborn screening study in Italy of more than 37,000 consecutive male neonates demonstrated an incidence of α-galactosidase A deficiency of 1 in 3100 [6]. In patients with end-stage renal disease on haemodialysis, studies have reported a prevalence from anywhere between 0.33% up to 1.2% [7,8]. One potential reason that the condition is under-recognised is that from the total number of cases of Fabry patients diagnosed, nearly half of them (46%) come from family screening [9]. This clearly demonstrates how difficult it is to find new cases / families and emphasising that when a new index of Fabry is diagnosed, screening the family is an important way of diagnosing previously undetected disease.

In general, hemizygous males are more severely affected than heterozygous females. In males, life expectancy is reduced by an average of 20 years [10] and in females by 15 years [11] with both being affected from an early age [12]. Death usually occurs due to renal, cardiovascular or cerebrovascular complications [1,10,11], with renal dysfunction being the main cause of death in men prior to the advent of renal failure requiring dialysis or transplantation [9].

Fabry nephropathy is characterised by variable levels of disease severity but with an overall rate of progression of chronic kidney disease (CKD) very similar to diabetic nephropathy, with evidence suggesting that all patients living into their 50s with classical Fabry disease will develop end-stage renal failure [9]. Accordingly, in addition to the aggressive treatment of hypertension, renoprotective measures should be introduced as soon as hyperfiltration and/or microalbuminuria are detected. Two enzyme replacement therapies (ERTs) have been shown to be effective in halting the progression of renal manifestations in patients with mild or moderate Fabry nephropathy and in slowing the progression of CKD in patients with advanced disease. Significant proteinuria and marked glomerulosclerosis (as with this patient) are the best predictors of progression of Fabry nephropathy despite ERT [13]. Consequently, ERT and nephroprotective measures should be started as soon as possible in male patients with the disease [14].

Given the importance of early initiation of treatment, timely and accurate diagnosis of Fabry disease is key. With this patient, as there were no clues to the presence of Fabry disease in the presentation, family history or clinical findings, making the correct diagnosis unknowingly rested on the interpretation of the needle biopsy. In general, this is not usually difficult however in this case which presented with advanced renal impairment; all 22 of the glomeruli obtained for light microscopy were globally sclerosed. Consequently the diagnosis rested completely on tissue collected and processed for EM. A semi-thin (0.5 μm) resin embedded survey section was stained with Toluidine blue to check for the presence of glomeruli before ultra thin sectioning for transmission EM. These steps showed the characteristic osmiophilic inclusions and Zebra bodies respectively (see Figure 2). A second histopathologist and member of the European Network of Fabry Pathologists confirmed these findings. A recent survey suggests that only half of institutions routinely collect tissue from native biopsies specifically for EM [15], confounded by an inadequate sampling rate. This case highlights the importance of EM in the evaluation of renal biopsies, underlining previous advice that this should be standard practice [16]. Indeed, in a review of laboratory practice in renal pathology, the evaluation of renal biopsy specimens without electron microscopy was regarded as negligent [17].

## Conclusion

In conclusion, this case highlights the importance both of Fabry disease as a differential diagnosis in patients with renal impairment in the context of multi-system disease and also of adequate tissue sampling for EM when performing native renal biopsies.

## Consent

The patient has given informed consent to the publication of this present case report and other anonymous reports on his clinical history. A copy of the written consent is available for review by the Editor-in-Chief of this journal.

## Competing interests

The authors declare that they have no competing interests.

## Authors’ contributions

JB identified the case for report, collated all the data and drafted the manuscript; JD prepared the histological specimens and their corresponding images; HB performed the cardiac ultrasound and prepared the images for the manuscript; WP ensured accuracy of the data and informed consent of the patient. All authors read, assisted with the editing of, and approved the final manuscript.

## Pre-publication history

The pre-publication history for this paper can be accessed here:

http://www.biomedcentral.com/1471-2369/13/73/prepub
